# Temporary loss of moral behavior in a patient undergoing chemotherapy with cisplatin - breaking bad

**DOI:** 10.1186/s12888-015-0386-0

**Published:** 2015-02-05

**Authors:** Kristian Barlinn, Hans Lehrach, Timo Siepmann, David Braeuer, Ulrich Buntrock, Norbert Sassim

**Affiliations:** Department of Psychiatry and Psychotherapy, Dresden-Friedrichstadt Municipal Hospital, Dresden, Germany; Department of Neurology, University Hospital Carl Gustav Carus Dresden, University of Technology Dresden, Dresden, Germany; Institute of Clinical Pharmacology, Carl Gustav Carus Medical Faculty, University of Technology Dresden, Dresden, Germany

## Abstract

**Background:**

Behavioral disturbances following chemotherapy with cisplatin are rare. Here, we report a patient with temporary loss of moral behavior in the setting of cisplatin-based chemotherapy for treatment of tonsillar cancer.

**Case presentation:**

A 66-year-old Caucasian male with no psychiatric or violent history was started on chemotherapy with cisplatin for treatment of tonsillar cancer. During the following weeks, the patient developed profound personality changes involving volatile emotions and impulsive aggression with verbal and physical assaults on others. Admitted to the hospital, the patient lacked any awareness that his behavior was wrong. Chemotherapy was discontinued and the patient was prescribed risperidone. Aside from mild cognitive impairment, comprehensive neuropsychological, neuroradiological and lab testing were unremarkable. Three weeks following cessation of chemotherapy, the patient had recovered to his original mental state and he was completely aware of his wrongdoing and social misconduct.

**Conclusion:**

Since neurotoxic effects of chemotherapeutics on the brain are not yet sufficiently elucidated, our case emphasizes that early signs of behavioral abnormalities in patients receiving chemotherapy should trigger comprehensive psychiatric evaluation and ongoing monitoring of the patients’ mental state.

## Background

Abnormal moral behavior resulting in social inappropriateness and aggressive conduct has been described as a result of structural damage to the frontal lobe of the brain [1-3]. We report on a patient with temporary loss of moral behavior with severe aggressive and violent outbursts in the setting of cisplatin-based chemotherapy for treatment of tonsillar cancer.

## Case presentation

A 66-year-old Caucasian male underwent curative tonsillectomy and ipsilateral radical neck dissection for right-sided tonsillar cancer (T2, N2b, cM0, G2; stage IVa). Four weeks after surgery, he was started on combined radiation therapy at a total dose of 60 Gy to the neck and chemotherapy with cisplatin (with standard schedule of 6 cycles á 40 mg/m^2^ = 73 mg, weekly).

During the following three weeks, the patient’s wife increasingly observed changes in her husband’s personality involving irritability, volatile emotions and impulsive aggression leading to verbal abuse and threats towards family members and neighbors (e.g., he delivered a letter to his neighbor including strong abusive language). The patient started to argue with his neighbor about proprietary rights of a water pump that has been shared for a long time. Ultimately he revealed physical aggression while cutting through the pipes of the water pump and chopping the house’s windowsills using an axe. After the fourth cycle of chemotherapy was completed, the situation escalated at home as his wife wanted to restrain him from driving his car after he had expressed suicidal thoughts in a state of agitation. He threatened to push her down the stairs unless she was willing to hand over the car keys. Finally their daughter called the police and emergency medical services and the patient was presented to the emergency department.

On initial mental state examination, the patient articulated himself clearly and was oriented to person, place and time. Initial testing of global cognitive functions did not reveal any gross impairment. The patient was calm and placid throughout the interview. When questioned about the preceding events, he trivialized them and revealed lack of any understanding that his behavior was wrong. Instead, he blamed his wife being responsible for his current situation and assured the admitting psychiatrist that he would avenge himself by giving his wife a sound beating once at home. His affect was euthymic with normal emotional reactivity while being inappropriate to the situation. The patient’s thought process and content were unremarkable. He denied any current suicidal ideation. Eventually he accepted admission to the acute psychiatric ward; however, he stated that his motivation was primarily the fact that this would spare him the daily drive to the hospital to receive ambulatory radiotherapy.

The patient did not have any other past medical or psychiatric history. He reported a history of chronic alcohol consumption (about two large beer per day), which he had ended after the diagnosis of tonsillar cancer 3 months ago with no occurrence of alcohol withdrawal symptoms. Drug history was insignificant. His wife assured that he had never been a violent or aggressive person in 42 years of marriage and that the current behavior was radically different. Brain contrast-enhanced computed tomography on admission revealed no significant findings. No abnormal findings were apparent on routine blood test (including blood alcohol level).

At first, the patient behaved cooperatively on the ward. The next day, however, he felt harassed by the volume of a fellow patient’s radio, so he angrily walked into the fellow patient’s room, intentionally destroyed the radio and assaulted her with his shoes and fists. He also attacked an attending nurse who came to intervene with his fists. Subsequently, the patient was restrained and, with obtained consent of the patient, intravenously treated with lorazepam. Further hospitalization commitment was ordered by court due to endangerment of others. Like the previous day, the patient appeared unrepentant and lacked any awareness of wrongdoing. Rather, he tried to rationalize his violent behavior by stressing that everyone feeling disturbed would have acted this way. The patient was started on neuroleptic treatment with risperidone 0.5 mg twice daily and lorazepam temporarily. The remaining two cycles of chemotherapy were not completed due to the unpredictability of the patient’s behavior; radiation therapy was continued as scheduled.

In the following, magnetic resonance imaging of the head showed a minor degree of general brain atrophy and slight chronic microvascular white matter changes. Cerebrospinal fluid examination including tests for infectious diseases (e.g., neurosyphilis) and biomarkers for Alzheimer’s Disease was unremarkable. No abnormal values were found on comprehensive blood tests including thyroid hormones and B vitamins. Although the patient scored within the normal range on both mini-mental state examination (MMSE, 28 points) and DemTect (16 points), the three-item clock-drawing test (CDT, 3 points) and Nuremberg Age Inventory (NAI) revealed a slight impairment in executive cognition. Specifically, he scored slightly below average in the NAI Labyrinth-, Number- and Word- Connection items (78, 83 and 84 points, respectively).

Over the course of the patient’s stay, the psychopathological condition improved constantly. Although still convinced that his conduct was justified, he was on good terms with the hospital staff and his fellow patients and increasingly behaved cooperatively and adequately. Therefore, the patient was put on home visit for stress vacation without any incidents and three weeks following cessation of cisplatin therapy, the patient’s wife confirmed that he had recovered to his original mental state. By that time, the patient was aware of his wrongdoing and regretted his recent social misconduct. He further emphasized that such conduct was not consistent with his personality. Of note, the patient did not experience any amnesia over the reported course. The patient was discharged four weeks after admission with continuation of risperidone and further treated by an ambulatory psychiatrist.

## Discussion

In this case report, a loss of moral behavior was noticed that was not related to an established psychiatric disorder or structural brain damage but occurred in temporal proximity to cisplatin-based chemotherapeutic treatment of tonsillar cancer. Discontinuation of chemotherapy and start of neuroleptic treatment may have reversed the patient’s psychopathological condition. Hence, our case highlights the importance of pharmacovigilance in chemotherapy with regard to psychiatric symptoms as these may affect compliance to treatment and, thus, long-term prognosis.

Recent reports have attributed certain encephalopathy syndromes (e.g., the posterior reversible encephalopathy syndrome) to cisplatin or carboplatin therapy causing severe neurological complications [4]; however, sole psychiatric disturbances following platinum-based chemotherapy seem rare. Since the introduction of platinum-based drugs in the 1970s for treatment of various types of cancer [5], only a few cases of new-onset psychotic or manic episodes were reported during treatment with platinum-based antineoplastic drugs [6-8].

The set of behavioral disturbances observed in our patient, namely disinhibition, impulsivity, inappropriate affect, emotional lability and poor judgment and insight was highly suggestive of a dysexecutive syndrome [1]. Although this syndrome has been formerly referred to as frontal lobe syndrome as dysexecutive symptoms are predominantly related to structural frontal lobe damage, research studies demonstrated that it may also involve cortical and subcortical regions outside the frontal lobe [1-3].

Neurological causes of dysexecutive syndrome comprise stroke, multiple sclerosis, tumor and dementia with permanent structural damages to the frontal lobe [1]. Although our patient did not show gross cognitive deficits indicated by standard neuropsychological testing, more in-depth tests (such as the three-item CDT and NAI) revealed slight impairment in executive cognition suggestive of mild cognitive impairment, which is commonly seen in, but not specific for, dysexecutive syndrome [9]. However, as changes in moral behavior are also frequently seen in mania, a manic episode was considered a differential diagnosis. In disconcordance with this differential, current psychopathology did not fulfill formal diagnostic criteria for an acute manic episode and history taken from our patient and his wife did not reveal any previous depressive or hypomanic/manic episodes [10]. Although we did not perform a specific test, an antisocial personality disorder or antisocial trait was not deemed likely either since the patient’s history did not reveal any corresponding symptoms prior to the treatment with cisplatin.

Alternatively, the patient’s behavioral disturbances might be explained by early dementia. After initial diagnosis of dementia, older patients are at an almost 10-times higher risk of developing a manic-like episode than older patients without dementia [11]. Conversely, mild cognitive impairment detected in our patient might be an early state of Alzheimer’s disease or other types of dementia and might therefore, pathomechanistically, constitute an synergism with cisplatin induced predominant frontal cerebral dysfunction leading to behavioral disturbances observed in this case [12]. This synergistic mechanism is also consistent with the previous observation that patients with higher cognitive reserves are able to compensate cognitive declining but this compensation can be compromised by exogenic noxa to the brain such as physical illness or certain drugs [13,14]. This noxa-induced decompensation of cognitive function in patients with subclinical dementia can be reversed by ending noxa exposition which is consistent with the return to normal behavior following cessation of cisplatin therapy in our patient [15].

Also, the presence of a synergistic pathomechanism with functional cerebral damage due to cisplatin therapy and preceding subclinical dementia in our patient might be consistent with the lack of reported cases of dysexecutive syndrome in cisplatin treated patients. However, mild cognitive impairment in our patient might have also resulted from chronic alcohol abuse (the latter in accordance with normal cerebrospinal fluid Alzheimer’s markers). Since the patient received radiotherapy treatment solely to the neck area with safety margins applied to spare surrounding, inter alia, brain tissues, we did not consider radiotherapy being related to the psychiatric outbreak in this case. This is underlined by the fact that radiation therapy was continued during hospitalization yet the patient improved constantly.

While peripheral sensory neurotoxicity mediated by DNA-cross links with positive charged molecules formed by hydrolytic aquation in dorsal root ganglion neurons is a common side effect of platinum-based antineoplastic drugs affecting almost one in two treated patients, cytotoxic effects of cisplatin to the central nervous system are not well elucidated [16,17]. One explanation for this apparent relative sparing of the central nervous system might be the characteristics of the compartmental distribution of the drug in vivo. In a study in 11 patients with ovarian cancer receiving cisplatin, the investigators observed equally high platinum concentrations in tumor, sural nerves, and spinal ganglia, but substantially lower platinum concentration in brain [18]. This loss of platinum on its way to the central nervous system might be caused by the reduced capacity of positive charged molecules formed by cisplatin aquation to cross the blood–brain barrier. Concordantly, following osmotic blood–brain barrier modification in an experimental setting in dogs, cisplatin was shown to induce disseminated cerebral foci of hemorrhagic necrosis and edema [19]. However, these focal lesions were also observed in other chemotherapeutic agents such as 5-fluorouracil and adriamycin and could therefore not be linked to a drug-specific pharmacodynamic mechanism.

While blood–brain-barrier induced peripheral-central concentration gradient of cisplatin in vivo might, in parts, explain the discrepancy of highly prevalent cisplatin induced peripheral nerve damage and low prevalent central adverse effects of cisplatin, it does not explain the occurrence of selective dysexecutive symptoms (with predominant frontal lobe pathology) following cisplatin administration as reported in this case. Our observation, viewed in conjunction with the current literature, might therefore suggest the possibility of an alternative coincident mechanism of cisplatin which causes predominant frontal cerebral neuron dysfunction in humans if the blood–brain-barrier has been passed to sufficient extent.

## Conclusion

Since neurotoxic effects of chemotherapeutics on the brain are not yet sufficiently elucidated, our case emphasizes that early signs of behavioral changes in patients receiving chemotherapy should trigger comprehensive psychiatric evaluation and ongoing monitoring of the patients’ mental state.

## Consent

The patient has given written consent for publication of this case report.

## References

[1] Godefroy O (2003). Frontal syndrome and disorders of executive functions. J Neurol.

[2] Raine A, Yang Y (2006). Neural foundations to moral reasoning and antisocial behavior. Soc Cogn Affect Neurosci.

[3] Fumagalli M, Priori A (2012). Functional and clinical neuroanatomy of morality. Brain.

[4] Zahir MN, Masood N, Shabbir-Moosajee M (2012). Cisplatin-induced posterior reversible encephalopathy syndrome and successful re-treatment in a patient with non-seminomatous germ cell tumor: a case report. J Med Case Rep.

[5] Amptoulach S, Tsavaris N (2011). Neurotoxicity caused by the treatment with platinum analogues. Chemother Res Pract.

[6] Matsunaga M, Onishi H, Ishida M, Miwa K, Araki K, Kaneta T (2012). Hypomanic episode during recurrent gastric cancer treatment: report of a rare case and literature review. Jpn J Clin Oncol.

[7] Ahmed N, Usmani S, Jabbour N, Hegde U (2011). Acute psychosis after paclitaxel infusion. Conn Med.

[8] Campbell BA, Panicker J (2011). New onset psychosis in an adolescent during treatment of testicular germ cell tumor. J Pediatr Hematol Oncol.

[9] Eslinger PJ, Damasio AR (1985). Severe disturbance of higher cognition following bilateral frontal lobe ablation: patient EVR. Neurology.

[10] American Psychiatric Association (2013). Diagnostic and statistical manual of mental disorders.

[11] Nilsson FM, Kessing LV, Sørensen TM, Andersen PK, Bolwig TG (2002). Enduring increased risk of developing depression and mania in patients with dementia. J Neurol Neurosurg Psychiatry.

[12] Bruscoli M, Lovestone S (2004). Is MCI really just early dementia? A systematic review of conversion studies. Int Psychogeriatr.

[13] Banga A, Gyurmey T, Matuskey D, Connor DF, Kaplan RF, Steffens DC (2013). Late-life onset bipolar disorder presenting as a case of pseudo-dementia: a case discussion and review of literature. Yale J Biol Med.

[14] Stern Y, Albert S, Tang MX, Tsai WY (1999). Rate of memory decline in AD is related to education and occupation: cognitive reserve?. Neurology.

[15] Nebes RD, Pollock BG, Houck PR, Butters MA, Mulsant BH, Zmuda MD (2003). Persistence of cognitive impairment in geriatric patients following antidepressant treatment: a randomized, double-blind clinical trial with nortriptyline and paroxetine. J Psychiatr Res.

[16] Mc Whinney SR, Goldberg RM, Mc Leod HL (2009). Platinum neurotoxicity pharmacogenetics. Mol Cancer Ther.

[17] Schindler C, Richter R, Kühndel K (1987). Central neurotoxic side effects following cisplatin therapy. Zentralbl Gynakol.

[18] Thompson SW, Davis LE, Kornfeld M, Hilgers RD, Standefer JC (1984). Cisplatin neuropathy. Clinical, electrophysiologic, morphologic, and toxicologic studies. Cancer.

[19] Neuwelt EA, Glasberg M, Frenkel E, Barnett P (1983). Neurotoxicity of chemotherapeutic agents after blood–brain barrier modification: neuropathological studies. Ann Neurol.

